# Response to Baverstock, K. Comments on Rithidech, K.N.; *et al.* Lack of Genomic Instability in Bone Marrow Cells of SCID Mice Exposed Whole-Body to Low-Dose Radiation. *Int. J. Environ. Res. Public Health* 2013, *10*, 1356–1377

**DOI:** 10.3390/ijerph10072735

**Published:** 2013-07-02

**Authors:** Kanokporn Noy Rithidech, Chatchanok Udomtanakunchai, Louise Honikel, Elbert Whorton

**Affiliations:** 1 Pathology Department, Stony Brook University, Stony Brook, NY 11974, USA; E-Mail: lmhonikel@hotmail.com; 2 Department of Radiologic Technology, Faculty of Associated Medical Sciences, Center of Excellence for Molecular Imaging, Chiang Mai University, Chiang Mai 50200, Thailand; E-Mail: oilchat@hotmail.com; 3 Institute of Human Infections and Immunology, Galveston National Laboratory, University of Texas Medical Branch, Galveston, TX 77555, USA; E-Mail: ewhorton@utmb.edu

We thank Dr. Baverstock [1] for his interest in reading our article and his time in writing his comments for our work [2]. We, however, respectfully disagree with his statement that we made “two category errors” associated with the assessment of the occurrence of “genomic instability” by determining the frequencies of delayed- or late-occurring chromosomal damage. Our disagreement is based upon the well-known fact that radiation-induced genomic instability (or delayed/late-occurring damage) can be manifested in many ways. These include late-occurring chromosomal damage, or mutations, or gene expression, or gene amplifications, or transformation, or microsatellite instability, or cell killing [3,4,5,6,7,8,9]. Such phenomena have been detected many cell generations after irradiation. We agree that genomic instability may well be the consequence of epigenetic changes. Another mechanism mentioned by Dr. Bavertock as being probably unlikely is the reversibility of damage. This potential may not be discarded off-hand, as Dr. Baverstock prefers to do. There is much reproducible evidence of adaptive protection that depending on absorbed dose precisely may reverse early damage, and damage appearing late may be due to some form of residual damage letting the cell become genetically unstable. In other words, the argument by Dr. Baverstock regarding upward or downward causation appears to be rather speculative and far from being settled. 

We stated very clearly in the Abstract section of our article that we determined the occurrence of genomic instability by the presence of late-occurring chromosomal damage, which is one of the known phenomenon associated with radiation-induced genomic instability. We clearly understand the meaning of genomic instability related to radiation exposure. We have used cytogenetic assays (both conventional and molecular cytogenetic methodologies) for studying biological effects of radiation (including radiation leukemogenesis) for about 20 years. Currently, we are also conducting experiments to study a link between radiation-induced genomic instability *in vivo* (assessed by the occurrence of delayed- or late-occurring chromosomal damage) and chronic inflammation, including aberrant DNA methylation patterns.

Kadhim *et al*. [10] was the first to describe the phenomenon of radiation-induced genomic instability using the chromosome aberration assay by detecting a significantly greater number of clonogenic survivors of exposed cells. Subsequently, several groups of investigators reported radiation-induced genomic instability, determined by other biological assays such as mutations, gene expression, or cell killing [3,4,5,6,7,8,9,11]. Numerous studies have reported the existence of genomic instability, as determined by the presence of delayed/late-occurring chromosome aberrations in the descendants of cells surviving radiation exposure [10,12,13,14,15,16,17,18,19]. Evidently, Dr. Baverstock’s argument is against not only our work but also the research of scientists around the world who are using cytogenetic and other assays (such as cell killings or cell transformation) to determine the occurrence of radiation-induced genomic instability. It should be noted that, as stated by Mothersill and Seymour [20] and Huang *et al*. [21], chromosome aberrations are the best-characterized end point of radiation-induced genomic instability.

It also is well known that there are two chromosome instability forms of genomic instability: non-clonal aberrations (such as chromatid breaks) and clonal aberrations (such as rearrangements) [5,14,20]. In our study, increases in these two types of chromo**s**ome aberrations were observed in bone marrow cells collected at 6 months after exposure of SCID mice to 0.1 or 1.0 Gy, **but not 0.05 Gy**, of ^137^Cs γ rays. Hence, Dr. Baverstock’s statement indicating that we made “a fundamental category error” associated with the assessment of the occurrence of genomic instability by determining the frequencies of late-occurring chromosomal damage is a scientifically unfounded criticism. Apparently, Dr. Baverstock’s argument against our work was based upon a narrow interpretation of radiation-induced genomic instability. Dr. Baverstock seems to pick and choose biological endpoints to determine the occurrence of genomic instability. For example, he chose epigenetics, as he cited his own article [22]. Of note, it is true that there is increasing evidence of a link between epigenetic events and radiation-induced damaged [23,24]. However, at this stage, Dr. Baverstock’s claim seems to be speculation and more work is needed to prove this point. Dr. Baverstock also selectively cited the paper by Falt *et al.* [25] which suggested that gene mutations play a role in radiation-induced genomic instability. This is not new because, as we pointed out earlier, the gene mutation assay also has been used to detected radiation-induced genomic instability. Further, one of the most relevant articles relating to clonal chromosome aberrations and genomic instability has been reported from this group of investigators two decades ago [16].

It also should be noted that previously reported data from other groups of investigators (using cytogenetic assays for determining the occurrence of radiation-induced genomic instability) [10,12,13,19,26,27] were derived from studies conducted with a combination of either: (*i*) *in vitro* irradiation and *in vitro* expression of genomic instability, or (*ii*) *in vivo* irradiation and *in vitro* expression of genomic instability, or (*iii*) *in vitro* irradiation and *in vivo* expression of genomic instability. In contrast, the approach used in our study [2] was *in vivo* irradiation/*in vivo* expression of genomic instability. 

Additionally, we would like to inform Dr. Baverstock about the correct mouse strains that we used in our previous work [28]. Dr. Baverstock indicated in his comments that “their early work at higher doses (0.1 and 1.0 Gy) on *the same strain of mouse* indicated that *de novo* chromosome aberrations were detected at 6 months post-irradiation”. This is an incorrect statement. The fact is that we used two other mouse strains, one with constitutively high (C57BL/6, a radioresistant strain) and one with intermediate levels (BALB/cJ, a radiosensitive strain) of the repair enzyme DNA-dependent protein-kinase catalytic-subunit (DNA-PKcs).

In that study, we reported no evidence of an *in vivo* induction of genomic instability (determined by delayed/late-occurring chromosomal damage) in bone marrow cells of BALB/cJ or C57BL/6 mice exposed to a single dose of 0.05 Gy of ^137^Cs γ rays. Taken together, a single dose of 0.05 Gy of ^137^Cs γ rays was incapable of inducing delayed/late-occurring chromosomal damage or genomic instability in bone marrow cells of three mouse strains with difference levels of endogeneous DNA-PKcs, *i.e.*, extremely low (SCID mice), intermediate (BALB/cJ mice), and high (C57BL/6J mice) levels. In contrast, the results from our studies demonstrated that a single dose of 0.1 or 1.0 Gy of ^137^Cs γ rays was capable of inducing genomic instability in bone marrow cells of exposed SCID and BALB/cJ, but not C57BL/6J mice. These findings indicate an influence of genetic background on radiosensitivity as previously reported by several investigators [29,30,31,32,33], and an important role of DNA-PKcs in DNA repair. Further, our findings in the mouse models support the observations of the linear relationship for human cancer that appears to hold to a dose of 0.1 Gy as suggested by several investigators [34,35,36,37].

Dr. Baverstock’s defense of the linear no threshold model (as stated in his comments that “epidemiological evidence from radiation exposed populations leaves little doubt……that the dose response is linear…..”) can be countered by several articles such as Averbeck *et al.* [38], Cohen [37], Cuttler [39], Dauer *et al.* [40], Feinendegen *et al.* [41], Jaworowski [42], Ogura *et al.* [43], Scott [44], and Tubiana *et al.* [45]. Regarding the f_0_ male mice injected with ^239^Pu cited by Dr. Baverstock, it is clear that the results from Ogura [43] demonstrated a reduction, **not an increase**, in mutation frequency by very low-dose gamma irradiation (500 µGy) after exposure of *Drosophila melanogaster*. Importantly, the results from the studies of low-dose radiation by Ogura and colleagues are contradictory to the LNT dose response theory that has been applied for radiation protection.

Overall, there is a large body of evidence demonstrating differences between biological responses to low (less than or equal to 0.1 Gy) and higher doses (more than 0.1 Gy) of radiation. It is now time to move forward beyond self-ideology. Each biological endpoint has its own merits and disadvantages. The best approach for future research is perhaps to use a combined testing paradigm as a metric for investigating both genetic and epigenetic events after exposure to low and high doses of radiation. A combination of different “omics” technologies would be useful. However, it is clear that in this constrained budgetary atmosphere, it is difficult (if not impossible) for a single laboratory to undertake the burden. Possibly, it is the time for global collaborative efforts. 
